# Acute reconstruction results in less sick-leave days and as such fewer indirect costs to the individual and society compared to delayed reconstruction for ACL injuries

**DOI:** 10.1007/s00167-019-05397-3

**Published:** 2019-02-14

**Authors:** Christoffer von Essen, Sebastian McCallum, Björn Barenius, Karl Eriksson

**Affiliations:** Department of Orthopaedics, Stockholm South Hospital, Karolinska Institutet, Stockholm, Sweden

**Keywords:** ACL, Acute, Outcome, Range of motion, Reconstruction, Costs

## Abstract

**Purpose:**

To compare the total number of sick-leave days caused by the knee injury from the day of injury and over the first year between acute (within 8 days) and delayed (6–10 weeks) anterior cruciate ligament reconstruction (ACLR) and also assess other clinical outcomes during this period.

**Methods:**

Seventy patients with an acute ACL injury and Tegner level of 6 or more were randomized to acute (within 8 days) or delayed (after 6–10 weeks) ACLR. Patient-reported outcomes; objective IKDC and manual stability measurements were assessed at 6 and 12 months. With data from the Swedish Social Insurance Agency (Försäkringskassan) information about the number of sick-leave days due to the knee injury over the following 12 months was collected and compared between the two groups.

**Results:**

Seventy-one percent received compensation for sick leave (26 in the acute versus 23 in the delayed group). The mean number of sick-leave days for the acute group was significantly lower (*M* = 56.9, SD = 36.4) compared to the delayed group (*M* = 88.5, SD = 50.2), *p* < 0.05. The acute group was also significantly stronger in flexion in both slow and fast angle velocities according to Biodex^®^. No other differences were found between the groups in other clinical assessments or in terms of associated injuries.

**Conclusion:**

Acute and delayed ACLR provided comparable clinical outcomes after 12 months. Acute reconstruction resulted in less sick-leave days and as such fewer indirect costs to the individual and society. These findings suggest that if patients requiring ACLR can be identified early and ACLR can be performed in the acute phase, socioeconomic costs can potentially be reduced by minimizing time off work.

**Level of evidence:**

II.

## Introduction

A rupture of the anterior cruciate ligament (ACL) is a serious knee injury most commonly affecting young, physically active persons. In both male and female athletes, ACL injury is the most frequent cause of permanent disability [1]. The incidence of ACL injury is around 7000 cases per year in Sweden with approximately 3500 ACL reconstructions (ACLR) performed annually [2].

ACL injuries may be managed non-operatively with physical therapy and activity modification or with surgical reconstruction of the ligament. Although conservative treatment can be successful in some patient groups [3], it has been proven less successful in physically active patients who compete or train at a high level [4–6].

The optimal timing of surgery has been debated [7–10], with surgical reconstruction traditionally delayed for at least 6 weeks due to evidence that earlier reconstruction may increase the risk of developing arthrofibrosis [11–13]. However, recent studies have demonstrated that with newer surgical techniques, surgery may be performed in the acute phase following the injury without significantly increasing the risk of complications [8–10].

An ACL injury is functionally disabling and may predispose the knee to further injuries, such as chondral and meniscal injury, while also contributing to early onset of degenerative changes in the knee [1, 14–18]. In addition to potentially reducing these risks, surgery in the acute phase following injury may facilitate earlier return to work, resulting in socioeconomic benefits to both society and the individual. There is currently conflicting evidence regarding the socioeconomic costs of acute versus delayed surgery, with two recent studies finding early ACLR to be more cost-effective than delayed surgery [19, 20] and another finding the opposite to be true [21].

In Sweden patients are entitled to paid sick leave if a doctor determines that work is not possible due to illness or injury, with up to 80% of income reimbursed. Costs are covered by the patient’s employer during the first 2 weeks of leave and subsequently by the government.

The primary aim of this study was to compare the total number of sick-leave days taken following ACL rupture in those undergoing acute and delayed reconstruction, as a means of measuring socioeconomic cost. The secondary aim was to compare early functional outcomes between the groups. It was hypothesized that an acute ACLR results in lesser sick-leave days without inferior patient-reported outcomes.

## Materials and methods

From 2006 to 2013, 2088 patients who had presented to the emergency department with an acute knee injury were followed up within 3 days at a knee clinic. Clinical examination and magnetic resonance imaging (MRI) were performed. If an ACL rupture was diagnosed and the patient consented to participation, they were assessed for inclusion in the study. The inclusion criteria for the study were: unilateral primary ACL injury in patients between 18 and 40 years of age with no previous knee injury to either leg, Tegner activity level [22] minimum 6, no additional meniscus or cartilage damage on MRI indicating the need for major acute meniscus or cartilage surgery, logistic availability to reconstruct the patient within 8 days of injury, no MCL-injury greater than grade 1 and no LCL-injury in need of surgery.

If all the pre-requisites were fulfilled, a research nurse performed randomization with the sealed envelope technique in the same session. Patient demographics are presented in Table 1. The patients were prospectively randomized to reconstruction of the ACL either within 8 days from injury or with delayed reconstruction after recovery of range of motion (ROM), within 6–10 weeks from injury. The patients randomized to delayed surgery received pre-operative physiotherapy to restore normal range of movement and to preserve muscle strength.

**Table 1:** Descriptives of the study population

	Total (*n* = 69)	Acute ACLR (*n* = 34)	Delayed ACLR (*n* = 35)	Sign
Age at inclusion mean ± SD	26.9 ± 6.1	27.7 ± 6.5	26.1 ± 5.7	n.s.
Gender: females *n* (%)	21 (31)	10 (30)	11 (31)	n.s.
Height cm mean ± SD	177 ± 9	177 ± 9	178 ± 9	n.s.
Weight kg mean ± SD	77 ± 11	76 ± 11	78 ± 12	n.s.
Smoker *n* (%)	4 (6)	2 (6)	2 (6)	n.s.
Highest education (*n* = 64) (%)				n.s.
High school/college	35 (55)	20 (65)	15 (45)	
University	29 (45)	11 (35)	18 (54)	
Main occupation *n* (%)				n.s.
Working	52 (75)	27 (79)	25 (71)	
Heavy manual labour	12 (23)	8 (30)	4 (16)	
Light manual labor	20 (38)	10 (37)	10 (40)	
Office work	17 (33)	8 (30)	9 (36)	
No compensation	3 (6)	1(4)	2 (8)	
Student	17 (25)	7 (21)	10 (29)	
Type of activity when injured *n* (%)				n.s.
Soccer	27 (38)	14 (41)	13 (37)	
Indoor floorball	16 (24)	6 (18)	10 (29)	
Alpine ski/snowboard	10 (15)	7 (20)	3 (8)	
Handball	5 (7)	1 (3)	4 (11)	
Wrestling/martial arts	3 (5)	3 (9)	0	
Gymnastics	2 (3)	2 (6)	0	
Ice hockey	1 (2)	0	1 (3)	
Am. football	1 (2)	0	1 (3)	
Badminton	1 (2)	0	1 (3)	
Basketball	1 (2)	0	1 (3)	
Dance	1 (2)	1 (3)	0	
Tennis	1 (2)	0	1 (3)	

Patient demographics at baseline are displayed as mean ± SD, number and percentage, respectively

At the time of inclusion and randomization the patients were evaluated regarding ROM (passive ROM measured with a goniometer and reported as a deficit in extension and flexion), instrumented laxity using the Rolimeter and thigh circumference measured 10 cm proximal to the proximal pole of the patella. Subjective and self-assessed IKDC [23], KOOS [24], Lysholm [22] and Tegner activity level were also evaluated.

In all clinical tests, the contralateral non-injured side was used as a reference.

### Surgical method

All patients underwent an arthroscopic ACLR with a hamstring tendon autograft. If the single semitendinosus tendon was not sufficient in length or thickness, the gracilis tendon was also harvested. In the beginning of the study the tibia was drilled first and transtibial drilling of the femur was used. The tibia angle was 45°–50° depending on the surgeon’s preferences, and a femur entry-point at 10 or 2 o’clock was preferred. Later in the study, due to the evolution of surgical technique at the time, the method for tunnel placement was changed. Drilling of the femoral tunnel was done through the anteromedial portal and the aim was to place the tunnel in the centre of the native footprint. The graft was fixed in the femur with an Endobutton continuous loop^®^ (Smith & Nephew, Inc., Andover, MA 01810, USA). In the tibia the graft was fixed with a metal interference-screw, RCI^®^ (Smith & Nephew, Inc., Andover, MA 01810, USA) or Soft Screw^®^ (Arthrex Inc., Naples, Florida 34108, USA). As an additional reinforcement the graft was fixed with an osteo-suture over a “bone-bridge” in the tibia. Injured menisci were sutured if an injury was discovered during the surgery.

### Rehabilitation

The rehabilitation was standardized to one physiotherapy centre and the same rehabilitation protocol was used for all patients. Closed-chain exercises and range of motion training was initiated within 1 week after the surgery. Open chain exercises were allowed after 6 weeks, running allowed after 14 weeks and resumption of sport activity after Biodex^®^ testing showed 90% strength in injured leg compared to the contralateral leg, but never earlier than 6 months.

### Postoperative follow-up

At 3 months ROM and circumference of the thighs were assessed by the patient’s physiotherapist. An independent physiotherapist not involved in the rehabilitation assessed the patients at 6 and 12 months.

The follow-up at 6 and 12 months included the same subjective scores as pre-operatively as well as ROM, circumference of the thighs and single leg hop. Isokinetic peak torque strength at 60°/s, 180°/s and 240°/s, and isometric torque strength at 60° and 180°, in both extension and flexion was measured with Biodex^®^ [25].

The number of sick-leave days claimed by each patient within the first 12 months from the injury was obtained from the government agency responsible for sick-leave payments (Swedish Social Insurance Agency-SSIA, http://www.forsakringskassan.se). Only sick-leave days based on a diagnosis of ACL injury were included.

The study was approved by the regional ethics committee at the Karolinska Institute, Stockholm Sweden (reference no. 2006/404-31/3/2008/1541-32).

### Statistical analysis

Statistical analysis was performed with the IBM SPSS 22.0 software package for Macintosh. Nominal variables were tested by the *χ*^2^ test or the Fisher exact test. Ordinal variables and non-normality distributed interval and ratio scale variables were evaluated by the Mann–Whitney *U* test. Student’s *t* test was used for normally distributed interval and ratio scale variables in independent groups. Longitudinal statistics were done with the paired-samples *t* test for normally distributed scale variables and the Wilcoxon signed rank test for ordinal and non-normality distributed scale variables. The tests were 2-sided. The results were considered significant at *p* < 0.05.

The study was originally designed to compare range of motion in the knee; hence students and patients who did not register for sick leave were not excluded. Furthermore, no power analysis regarding differences in days of sick leave was performed prior to study commencement, a post hoc power analysis using G*Power 3.1.9.2 (Franz Paul, Kiel, Germany) was used to determine the power of the present study. Based on number and the distribution of sick-leave days at 1 year in the study population, an effect size of 0.73 was calculated, with *α* set at 0.05 and 2-sided tests, the power of the study was calculated to 71%.

## Results

**Fig. 1:**
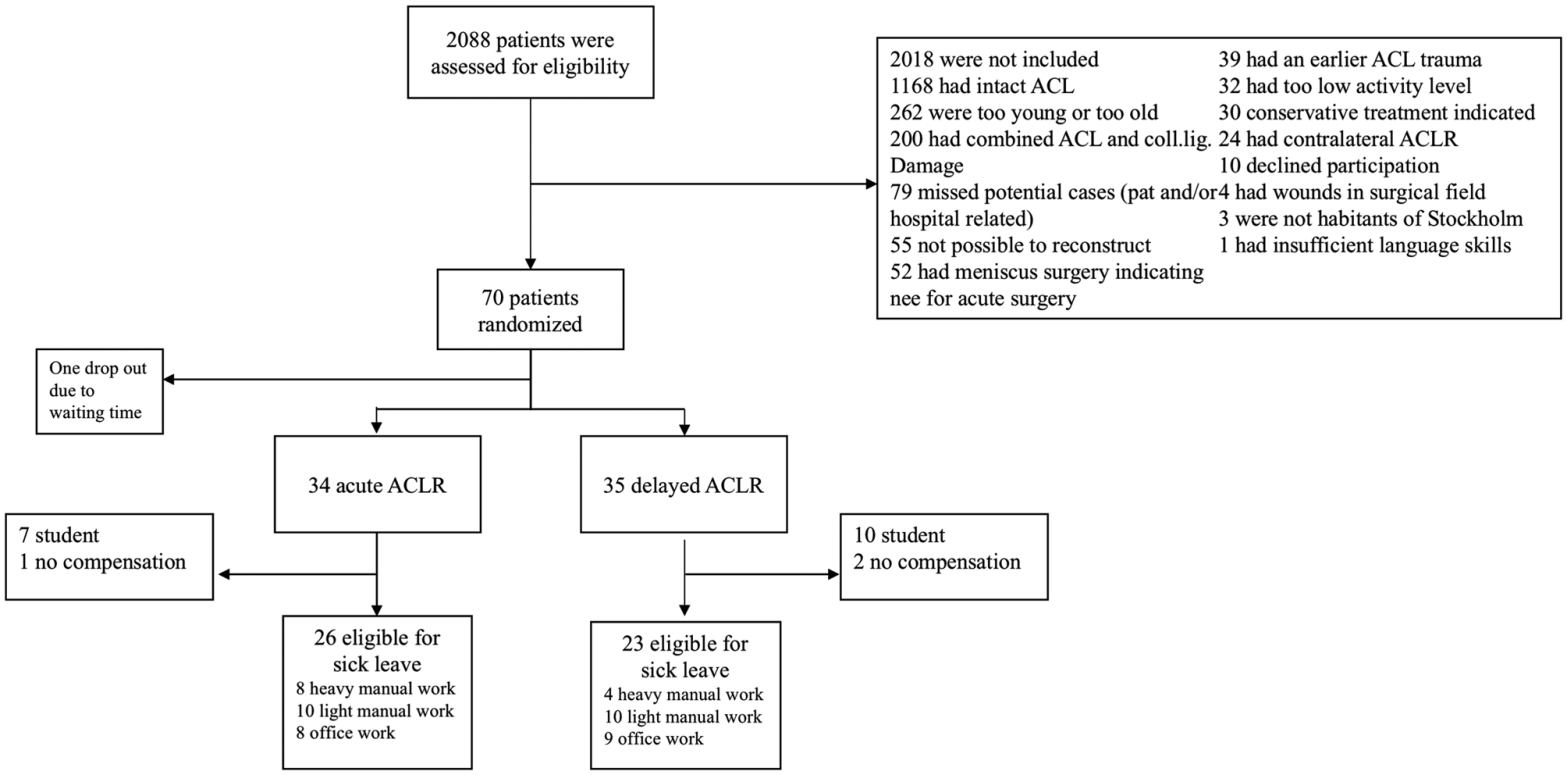
Enrollment, randomization and sick-leave compensation of subjects

Seventy patients were included, with 35 patients randomized to early ACLR and 35 patients to late reconstruction. One patient from the delayed group dropped out before surgery due to personal reasons, a second was excluded as they were unable to participate in follow-up according to the study protocol (Fig. 1).

Demographic data of the study groups are displayed in Table 2. At the 12-month follow-up, 66 (94%) patients were available for the clinical follow-up. Three from the acute and one from the delayed group was missing regarding clinical results. The only significant difference between the groups at the time of surgery was the time between injury and reconstruction.

**Table 2:** Demographics

	Acute ACLR (*n* = 34)	Delayed ACLR (*n* = 35)	Sign.
Time injury-recon (d ± SD)	5 ± 2	55 ± 8	< 0.01
OP time (min ± SD)	93 ± 20	83 ± 18	n.s.
Graft diameter (mm ± SD)	8.8 ± 0.8	8.6 ± 0.8	n.s.
Additional injury [*n* (%)]	21 (66)	15 (47)	n.s.
Medial meniscus [*n* (%)]	7 (21)	2 (6)	n.s.
Lateral meniscus [*n* (%)]	13 (39)	10 (29)	n.s.
Sutures [*n* (%)]	3 (9)	1 (3)	n.s.
Cartilage inj. [*n* (%)]	10 (29)	4 (11)	n.s.
Transtibial technique [*n* (%)]	12 (35)	10 (29)	n.s.
Anteromedial technique [*n* (%)]	22 (65)	25 (71)	n.s.

Patient demographics at baseline for patients who underwent ACLR are displayed as mean ± SD, number and percentage, respectively. Statistically significant (*p* < 0.05) values were only seen for the time from injury to reconstruction

### Sick-leave days

All patients were assessed regarding sick leave during the first year. Seventeen (25%) patients out of 69 were students without registered compensation for sick leave. An additional 3 (4%) patients were not students, but were not registered for any compensation for sick leave from work; these patients were predominantly self-employed and did not claim any sick leave compensation. 49 out of 69 (71%) patients received compensation for sick leave, 26 in the acute group and 23 in the delayed group. No differences were seen in regard to occupations involving heavy or light manual labor; Table 1.

**Fig. 2:**
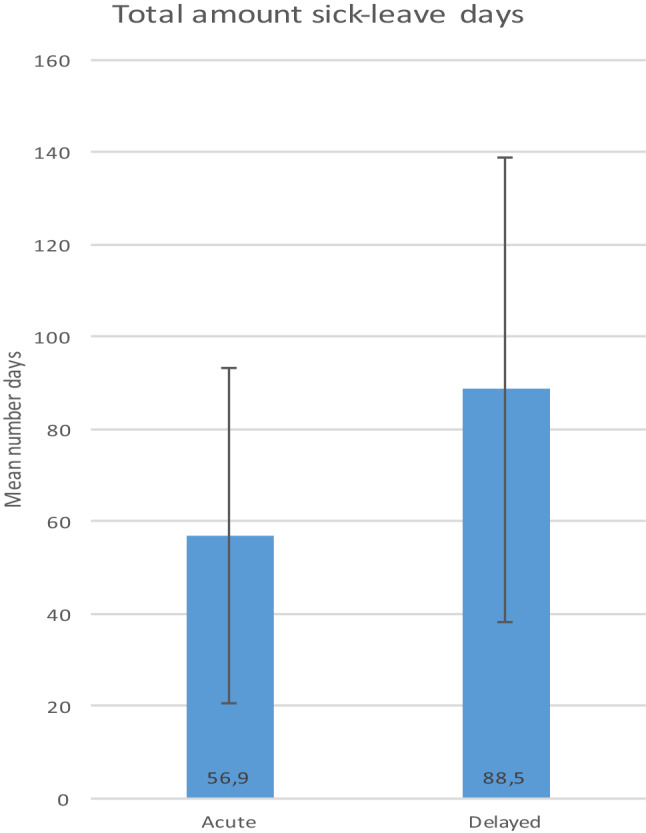
Total amount sick-leave days

Total amount of days’ sick leave by study group are presented in Fig. 2. The mean number of sick-leave days for the acute group was significantly lower (*M* = 56.9, SD = 36.4) compared to the delayed group (*M* = 88.5, SD = 50.2), *p* < 0.05.

Distribution of sick-leave days differed between the groups, the acute group had one continuous period, while the delayed had either one continuous or two separate periods depending on the physical demands of the occupation. The delayed group was on sick leave for a mean of 23 days before reconstruction.

**Fig. 3:**
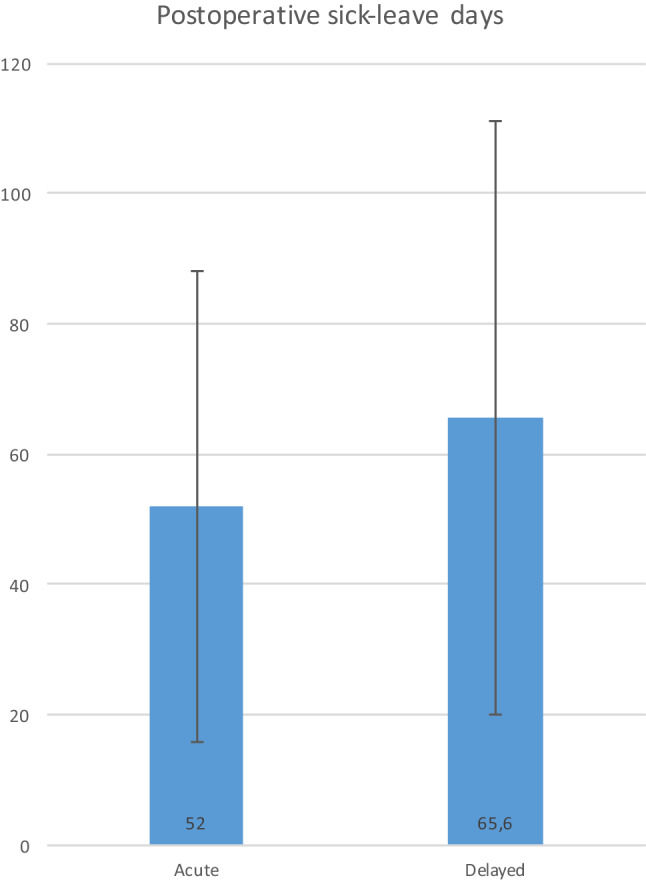
Postoperative sick-leave days

On average the acute group also had fewer days for recovery after surgery, but not statistically different (*M* = 52.0 SD = 36.1) compared to the delayed group (*M* = 65.6, SD = 45.5), *p* = n.s.(0.25) (Fig. 3).

### Patient-reported outcome

**Fig. 4:**
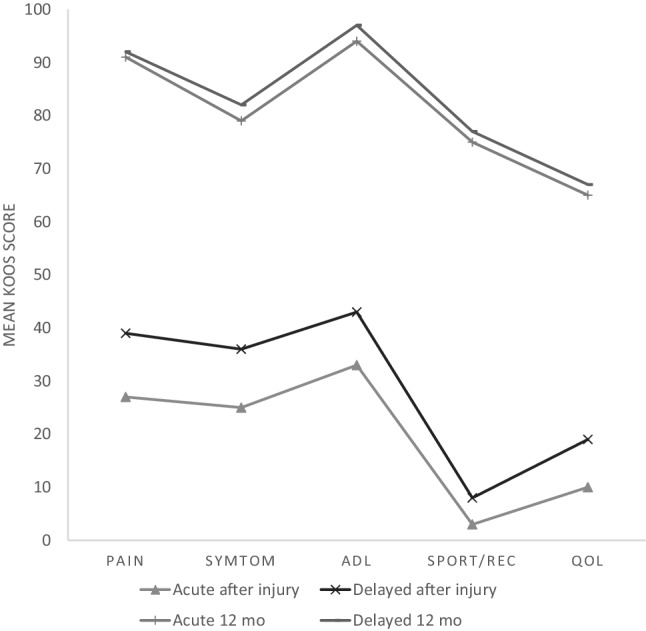
Mean KOOS scores with significant changes after injury to 12 months, but no significant difference between the groups at any time-point

After the injury, the acute group was less affected in the KOOS subscales ‘pain’ and ‘quality of life’. After 12 months, the KOOS were similar in the groups (Fig. 4), with significant changes after injury to 12 months, but no significant difference between the groups at any time-point. Both groups had improved Tegner and Lysholm scores from inclusion to the 12-month follow-up (Table 3).

**Table 3:** Patient-reported outcomes, knee laxity measures and strength at 12 months

	Acute ACL reconstruction (*n* = 31–34)	Delayed ACL reconstruction (*n* = 32–34)	*p* value
Patient-reported outcomes at 12 months			
Lysholm mean (SD)^a^			
Inclusion	32 (21.5)	43 (26.2)	n.s
6 months	76 (16.2)	79 (15.2)	n.s
12 months	87 (18)	88 (17)	n.s
Tegner median (range)^b^			
Before injury	8 (6–10)	9 (5–10)	n.s
At inclusion	0 (0–6)^c^	0 (0)	0.001
6 months	4 (1–9)	4 (0–9)	n.s
12 months	6 (1–9)	6 (0–9)	n.s
Instrumented knee laxity			
Rolimeter mean mm (SD)	2.0 (1.4)	1.9 (1.2)	n.s
Mean degrees (SD) w ref CL limb			
Extension deficit	2 (2.1)	3 (3.3)	n.s
Flexion deficit	1.8 (2.2)	3.2 (3.4)	n.s
No (%) normal Pivot shift test^d^	30 (94)	29 (88)	n.s
IKDC Score *n* (%)^e^			
6 months			
AB	27 (82)	24 (71)	n.s
CD	6 (18)	10 (29)	
12 months			
AB	26 (84)	27 (80)	n.s
CD	5 (16)	7 (20)	
Functional strength			
Thigh deficit circ. 10 cm above patella diff in cm ref CL	0.8	1.24	n.s
One-leg hop *n* (%)^f^			
> 90	23 (74)	21 (63)	n.s
76–89	6 (19)	8 (24)	
50–75	1 (3)	2 (6)	
< 50	1 (3)	2 (6)	
Muscle strength Biodex^®^			
Peak torque % ref to CL limb^g^			
Ext. isokinetic			
60°/s	90.2	82.9	0.08
180°/s	91.7	86.5	n.s
Flex. isokinetic			
60°/s	95.6	88.3	0.005
180°/s	96.7	90.2	0.01

*ACL* anterior cruciate ligament, *CL* uninjured contralateral limb

^a^Score range from 0 to 100, with higher scores indicating better results

^b^Assesses activity level with specific emphasis on knee; scores range from 1 (least strenuous activity) to 10 (high knee demanding activity on professional sports level) 15

^c^One patient answered 6 at inclusion, misunderstood guidelines

^d^Assesses rotational stability of knee at rest result range from 0 (normal stability) to 3 (severely increased instability)

^e^Assesses knee function, AB normal or near normal, CD abnormal

^f^Result indicates if the patient is ready to return to play. To pass, the involved leg must measure at least 90% of the distance compared to the uninvolved leg

^g^Comparison of extensor and flexor torque deficits collected for isometric Biodex, displayed as mean percentage with reference uninjured CL set at 100

The overall objective, IKDC as well as manual laxity measurements using pivot shift and Rolimeter did not demonstrate any statistically significant differences (Table 3).

Similar ROM between the groups was found at 12 months measured at the hospital unit (Table 3).

No differences were found between the groups in the one-leg hop test or thigh muscle atrophy. At 12 months after reconstruction there were more patients cleared to return to sports in the acute group, however there was no statistical difference.

The muscle strength peak torque in percent of uninjured limb was significant higher for the acute group (Biodex^®^ test).

## Discussion

The major finding of this study was that a reduction in indirect costs (as defined by lost wages and sick-leave days) was seen in the acute ACLR group. These results support the notion that early ACL reconstruction could be more cost-effective than delayed surgical treatment strategies as reported in several studies [19, 20, 26, 27].

In both groups, there was a significant improvement in self-reported quality of life after the injury to 1 year after surgery. There was no difference between the groups with respect to the IKDC score [23], with 83% of the participants in the acute group scoring either A or B compared to 80% in the delayed. There were no significant differences in measurements of knee laxity and knee ROM, suggesting that the benefits of acute surgery are primarily seen in the early phase after the surgery and that good results can be achieved after 1 year regardless of timing. There was no significant muscle atrophy of the thigh muscles compared to the contralateral leg in either group at the 12 months follow-up. There was however a significant difference in knee flexion strength, with an advantage for acute surgery, indicating that there is a potential for faster muscle recovery with acute surgery.

The findings in this study are supporting the conclusion of Herbst et al. that ACLR within 48 h is preferable in highly active patients to avoid unnecessary delay for return to full activity [9]. Our findings are also consistent with those of Maher et al. who found a greater improvement of QALY with ACLR compared to structured rehabilitation alone and concluded that it is the unstable knee that costs the society due to the loss of wages, productivity and disability.

In contrast to the results in this study, Frobell et al. found a lesser QALY improvement after ACLR, and therefore found ACLR to be less cost-effective. They concluded that early reconstruction was not superior to initial nonsurgical treatment with optional delayed reconstruction and stated that early reconstruction did not have any economic benefits [21, 28]. A consideration regarding this conclusion is the number of patients in the study that underwent reconstruction in the optional delayed group. At the 5-year follow-up 51% had undergone delayed reconstruction and these patients required significantly more meniscus procedures compared to those who underwent early reconstruction [29]. The study by Frobell et al. also excluded sick-leave costs that did not extend over 14 days.

The differences in costs between the two strategies are affected by several variables and can be measured in different ways. As such, using sick days as a proxy for socioeconomic costs has its limitations and may not completely reflect socioeconomic costs. However, this study supports our assertion that a change in the timing of ACL surgery, when indicated, could reduce the socioeconomic costs to society resulting from ACL injury.

It is not always obvious which patients should undergo early reconstruction and which patients will achieve a good outcome with conservative treatment. A patient with high functional demands and risk for giving way, who is not prepared to adjust activities and has an unstable knee immediately post-injury, is generally better treated with acute ACLR. A low-demand patient with below average risk of symptomatic instability can undergo rehabilitation first and undergo delayed ACLR should instability arise. The challenge is often determining which treatment is optimal in patients who do not neatly fit into these two groups, balancing the risks of unnecessary surgery for those who could successfully be managed conservatively against the risks and costs of treatment delay for those who go on to require surgery.

The major strength of this study is the prospective, randomized design with four experienced surgeons performing all of the ACL reconstructions with the same surgical technique. Furthermore, one centre with the same postoperative rehabilitation protocol was used in both groups. Another strength is that the study population was selected from a highly motivated patient category with an activity level that justifies reconstruction and a strict definition of the time points for surgical intervention based on the time from injury to surgery in the acute group was used. Furthermore, the loss to follow-up rate was negligible (*n* = 1) regarding data on sick-leave.

The results of this study should however be interpreted in light of some limitations. Firstly, the pre-study power analysis was made to detect differences in range of motion and not days of sick leave. However, the differences between the groups are large which increases the likelihood of the results being true even with a post hoc power analysis of 70%. Secondly, despite there being several other factors that affect the total cost of ACLR, we have only assessed the number of sick-leave days as a proxy for socioeconomic costs. We also did not have a nonoperative counterpart for comparison and our study is only focused on individuals with at least a moderate activity level.

As for clinical relevance, this study demonstrates that acute ACLR can be performed safely in highly active patients and ensure an earlier return to work and full activity.

## Conclusion

Acute and delayed ACLR provided comparable clinical outcomes after 12 months. Acute reconstruction resulted in less sick-leave days and as such fewer indirect costs to the individual and society. These findings suggest that if patients requiring ACLR can be identified early and ACLR can be performed in the acute phase, socioeconomic costs can potentially be reduced by minimizing time off work.
